# Delayed Resolution of Feeding Problems in Patients With Congenital Hyperinsulinism

**DOI:** 10.3389/fendo.2020.00143

**Published:** 2020-03-18

**Authors:** Chris Worth, Caroline Hall, Sarah Wilson, Niamh Gilligan, Elaine O'Shea, Maria Salomon-Estebanez, Mark Dunne, Indraneel Banerjee

**Affiliations:** ^1^Department of Paediatric Endocrinology, Royal Manchester Children's Hospital, Manchester, United Kingdom; ^2^Therapy and Dietetic Department, Royal Manchester Children's Hospital, Manchester, United Kingdom; ^3^Faculty of Biology, Medicine and Health, University of Manchester, Manchester, United Kingdom

**Keywords:** congenital hyperinsulinism, feeding, feeding problems, feed aversion, natural history, lesionectomy

## Abstract

**Background:** Congenital Hyperinsulinism (CHI) is the most common cause of recurrent and severe hypoglycaemia in childhood. Feeding problems occur frequently in severe CHI but long-term persistence and rates of resolution have not been described.

**Methods:** All patients with CHI admitted to a specialist center during 2015–2016 were assessed for feeding problems at hospital admission and for three years following discharge, through a combination of specialist speech and language therapy review and parent-report at clinical contact.

**Results:** Twenty-five patients (18% of all patients admitted) with CHI were prospectively identified to have feeding problems related to sucking (*n* = 6), swallowing (*n* = 2), vomiting (*n* = 20), and feed aversion (*n* = 17) at the time of diagnosis. Sixteen (64%) patients required feeding support by nasogastric/gastrostomy tubes at diagnosis; tube feeding reduced to 4 (16%) patients by one year and 3 (12%) patients by three years. Feed aversion resolved slowly with mean time to resolution of 240 days after discharge; in 15 patients followed up for three years, 6 (24%) continued to report aversion. The mean time (days) to resolution of feeding problems was lower in those who underwent lesionectomy (*n* = 4) than in those who did not (30 vs. 590, *p* = 0.009) and significance persisted after adjustment for associated factors (*p* = 0.015).

**Conclusion:** Feeding problems, particularly feed aversion, are frequent in patients with CHI and require support over several years. By contrast, feeding problems resolve rapidly in patients with focal CHI undergoing curative lesionectomy, suggesting the association of feeding problems with hyperinsulinism.

## Introduction

Congenital Hyperinsulinism (CHI) is the most common cause of severe and recurrent hypoglycaemia in childhood with an incidence between 1:2500 and 1:50000 (1). There is a high risk of hypoglycaemia related brain injury in both transient and persistent forms of the disease (2). Broadly there are two types of CHI: focal CHI in which a single isolated region of the pancreas causes hyperinsulinism or diffuse CHI where all parts of the pancreas are hyperinsulinaemic. For both focal and diffuse CHI, patients often require a prolonged admission in hospital, normally combined with intensive medical treatment and often supraphysiological carbohydrate requirements to maintain stable blood glucose. Many patients require nasogastric tube (NGT) or gastrostomy feeds to deliver enriched nutrition. Nasogastric tube would be selected in the first instance as a temporary solution to provision of enteral nutrition and this would be converted to gastrostomy, after discussion with families, if the clinical team felt non-oral enteral feeding would be required for a prolonged period. When enteral nutrition fails, higher concentration dextrose administered by parenteral nutrition (PN) may be required. The use of non-oral feeding regimens combined with medications which induce nausea and the interruption of normal feeding milestones are contributing factors which place infants with CHI at risk of feeding problems (3).

Feeding problems have long been recognized as a common problem in CHI (4) and were identified in a cohort of patients undergoing subtotal pancreatectomy (5). Feeding problems with vomiting, sucking and swallowing difficulties and feed aversion in isolation or in combination have been estimated to affect between 31 and 45% of CHI patients worldwide (4–7). It is acknowledged that feeding problems are a significant source of stress and concern for families with CHI (3, 4) as well as being an obstacle to the administration of sufficient quantities of carbohydrate required for the successful treatment of hypoglycaemia and the prevention of neuroglycopaenic brain injury.

Our group has previously found that severity of CHI is a strong predictor for the prevalence of feeding problems (7). Feeding problems can persist beyond resolution of hypoglycaemia in patients with diffuse forms of CHI (6, 7) with variations between those undergoing surgery and those treated medically (4). The long-term outcome of feeding problems in CHI has not been characterized. There are no longitudinal follow-up studies to investigate predictive factors for resolution/persistence of feeding problems. We have investigated a 2-year cohort of patients with feeding problems and CHI with the following objectives:

1. Investigate feeding problem types and rates of persistence in children with CHI over a three year follow up period.
2. Identify factors that predict resolution of feeding problems within those three years.

We did not aim to address predictive factors for development of feeding problems as our group has addressed this previously (7).

## Methods

All patients with CHI admitted to Royal Manchester Children's Hospital (RMCH) were reviewed by a specialist Speech and Language Therapist and a Dietitian as part of a multidisciplinary team approach to specialist clinical management. Diagnosis of CHI was made on the basis of criteria described elsewhere (8) but focused on the measurement of detectable levels of insulin (>8 pmol/L) at the time of hypoglycaemia (plasma glucose <2.6 mmol/L) with associated features such as hypoketosis and high glucose requirement (> 8 mg/kg/min). Genetic testing was performed in those patients unresponsive to diazoxide and in those persistently requiring more than 5 mg/kg/day. Rapid *ABCC8*/*KCNJ11* mutation testing by Sanger sequencing was undertaken initially, followed by a targeted next generation panel of 14 genes (ABCC8, KCNJ11, GLUD1, HADH, GCK, HNF4A, HNF1A, SLC16A1, CACNA1D, KDM6A, KMT2D, PMM2, INSR1, and TRMT10A) known to cause isolated or syndromic CHI (9).

From January 2015 until December 2016 (two years), all patients with CHI with identified feeding problems were entered prospectively onto a database by the specialist Speech and Language and Dietetics team. Demographic and clinical data including types of feeding problems and frequency of persistence were collected prospectively.

The database was updated through both planned and unplanned clinic/inpatient reviews following discharge after the initial hospital admission. The principle route of feeding, additional enteral nutritional routes and resolution/persistence of feeding problems were documented at six-month intervals until three years post discharge. Neurodevelopmental evaluation was performed as part of MDT assessment at each clinical contact by an experienced clinical psychologist. Feeding problems were classified as problems related to sucking, swallowing, vomiting, reluctance, and feed aversion as per previous studies on feeding problems by our group (7). Data stored in this database was corroborated with information derived from electronic medical records and additional telephone research clinics.

Vomiting was diagnosed as a feeding problem if >50% of food contents were brought up for at least 50% of feeds. Sucking problems were diagnosed objectively by a specialist Speech and Language Therapist which, in this cohort, most commonly included: inability to maintain lengthy suck bursts within suck-swallow breathe sequence, poor development of sucking due to missed critical time periods in relation to the establishment of oral feeding, and early refusal of latch and consequent initiation of suck-swallow breathe pattern. In contrast, swallowing problems were identified where there was clear evidence of potential signs of aspiration on clinical assessment.

When sucking and swallowing problems were persistent enough to prevent safe and effective enteral feeding, an NGT was passed for administration of feeds. NGT was removed once children were orally fed for over 24 h or a gastrostomy tube had been inserted. Other reasons for insertion of NGT or gastrostomy included supraphysiological carbohydrate requirement (not achievable via oral feeding) and feed aversion. For patients requiring persistent NGT feeding, a surgical gastrostomy tube insertion was considered based on a clinical decision by the multidisciplinary team in discussion with the family and individualized to the patient's need. Feed reluctance and feed aversion were diagnosed by a specialist Speech and Language Therapist incorporating the SOS Approach to Feeding Toolkit (https://sosapproachtofeeding.com/) with the difference between the two relating to severity and demonstrated behavior on attempted feeding.

Neurodevelopmental problems were classified as none, some or severe (2). Some neurodevelopmental problems were defined as clinical concerns about delayed development. Severe neurodevelopmental problems were defined as above with the additional features of seizures or cortical visual impairment.

All data were analyzed using IBM SPSS Statistics 25. Data were analyzed for normality using the Shapiro-Wilk test and differences between continuous variables were assessed using the Mann-Whitney *U* or Kruskal-Wallis tests as all data were non-normally distributed. Cox regression analysis was used to assess the impact of various clinical variables upon the resolution/persistence of feeding problems and feed aversion over time. Spearman Rank tests were used to assess for correlations between continuous variables. The study was covered by local research ethical approval (REC/H1010/88).

## Results

### Prevalence of Feeding Problems

During the study period, 138 patients were admitted to RMCH with CHI. Of these, 25 (18%) (12 female) patients were prospectively identified as having a feeding problem. Most patients presented early, with a median (range) age at diagnosis of 3 (1–324) days. Those with focal CHI presented later than diffuse CHI with a median (range) age of 87 (3–150) days vs. 2 (1–324) days at presentation (*p* = 0.03). CHI mutations were identified in 14 (56%) patients with 13 affecting the K_ATP_ channel (*ABCC8/KCNJ11*) and one being a novel glucokinase (*GCK*) mutation. There were six (24%) patients with focal CHI, with the rest having diffuse CHI. Four patients with focal CHI had curative lesionectomies. In two patients with focal CHI, the lesions identified by 18-fluoro-dopa PET-CT scanning were not anatomically located by frozen section histopathology; conservative medical treatment was continued in these patients. For those with diffuse CHI, eight (32%) patients required a subtotal pancreatectomy for control of hypoglycaemia. Neurodevelopmental problems were present in eight (32%) patients and were severe in 4 (16%) patients. Brain magnetic resonance scans were performed in six of the patients with neurodevelopmental problems based on clinical need and demonstrated findings consistent with and consequent to neonatal hypoglycaemia, such as cystic encephalomalacia and gliosis.

Hypoglycaemia was severe with a mean Glucose Infusion Rate (GIR) of 14 mg/kg/min and 15 (60%) patients requiring additional intravenous glucagon to maintain glycaemic stability. All children were initially treated with diazoxide but 18 (72%) patients were not responsive and required octreotide for the management of hypoglycaemia. Eight (32%) patients required the use of Parenteral Nutrition (PN) with high concentration dextrose while in hospital. The median (range) length of stay in hospital was 78 (21–258) days.

### Types of Feeding Problems

The most common feeding problem identified at the time of initial hospital admission was vomiting, present in 20 (80%) patients. Sodium alginate containing compounds (*n* = 13), ranitidine (*n* = 15), omeprazole (*n* = 10), and domperidone (*n* = 4) were used to treat presumed gastro-oesophageal reflux to improve vomiting. Vomiting resolved in 5 (25%) of patients by the time of discharge from hospital. In the rest, vomiting resolved at a median (range) 150 (6-1705) days after discharge from hospital.

Sucking and swallowing problems were less common, present in six (24%) and two (8%) patients respectively with time from discharge to resolution of 192 (1–1705) and 643 (23–1310) days respectively. Eight (32%) patients demonstrated reluctance with food and/or had an element of texture sensitivity; in these patients, time from discharge to resolution was prolonged at 1145 (125–2037) days.

**Figure 1:**
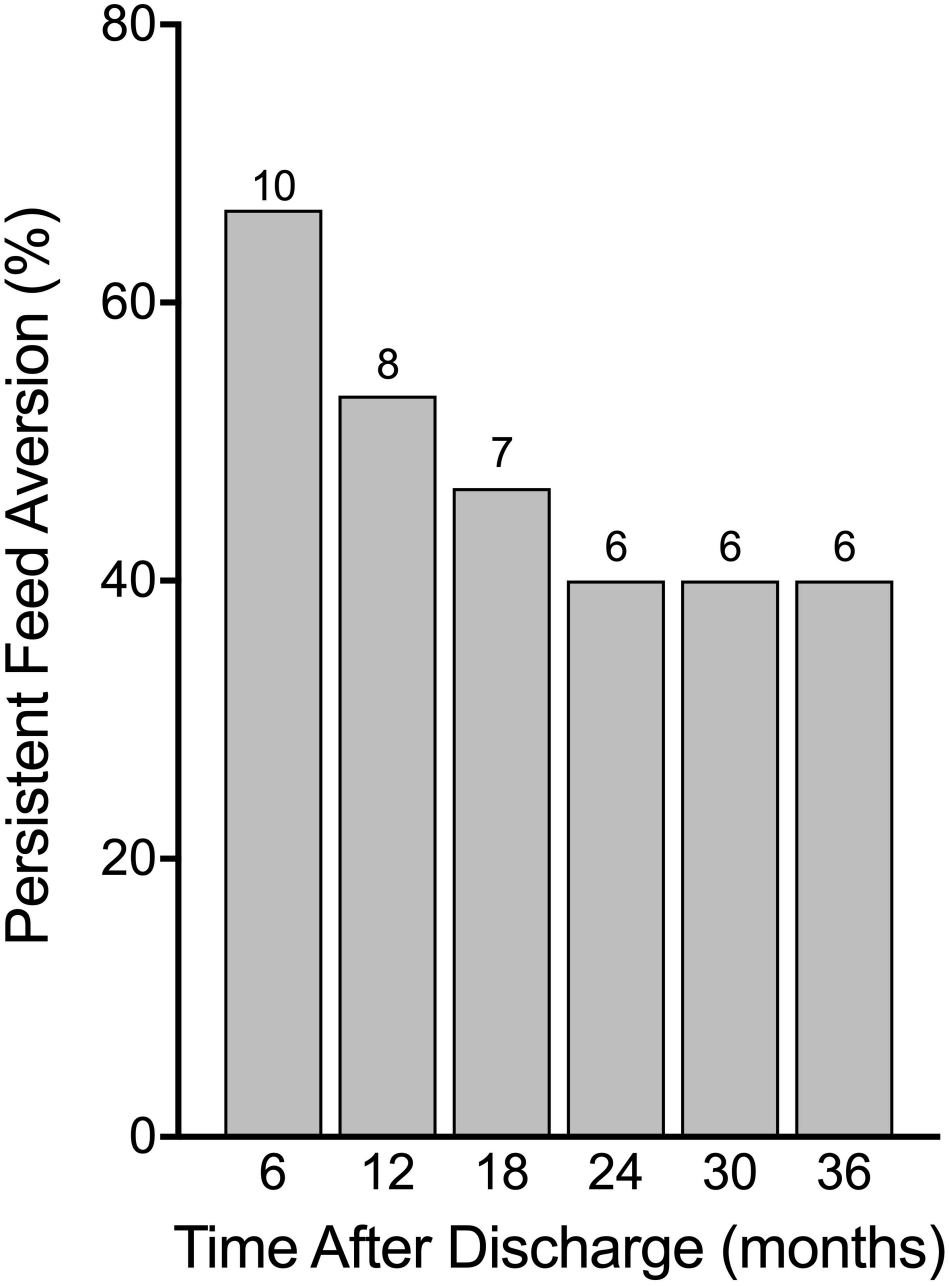
Persistence of feed aversion in percentage of those diagnosed with aversion while an inpatient with absolute numbers above bars. While feed aversion did tend to improve after discharge, after 24 months following discharge, no improvements were seen and feed aversion persisted.

Feed aversion was documented in 17 (68%) patients with a widely variable time from discharge to resolution of 240 (16–2037) days. Full follow-up data was available for 15 of the 17 patients with feed aversion, while data was incomplete in the other two patients. In regular follow up assessment, the incidence of feed aversion decreased only gradually over time (Figure 1). At two years' post discharge, six (40%) patients continued to have feed aversion. Beyond two years, there was no reduction in the rate of feed aversion over the following year (Figure 1).

**Figure 2:**
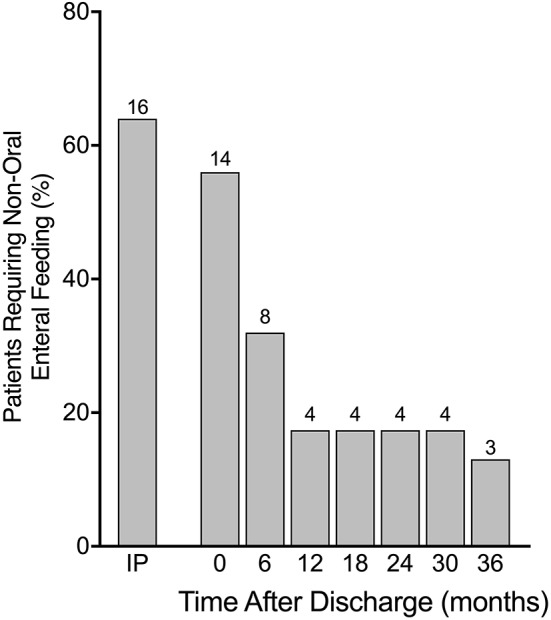
Requirement for non-oral enteral feeding in percentages from time as inpatient (IP) until 36 months after discharge with absolute numbers above bars. Many patients required non-oral feeding via either nasogastric tube (NGT) or gastrostomy while an inpatient with significant reduction in frequency by 12 months post hospital discharge and then a plateauing of improvement until 36 months.

During the initial period of admission, 16 (64%) patients required some form of non-oral enteral feeding (6 via NGT, 10 via gastrostomy). The frequency decreased slightly by the time of discharge to 14 (56%) patients (4 via NGT, 10 via gastrostomy). By six months post discharge only eight (32%) patients required non-oral enteral feeding (4 via NGT, 4 via gastrostomy) and by 12 months this number was only four (16%) (all via gastrostomy). Over the following two years only one patient was able to stop gastrostomy feeds, leaving three (12%) patients still requiring non-oral enteral feeds at three years post discharge (Figure 2). Of those patients requiring gastrostomy feeds at 12 months post discharge, feed aversion was present in three patients in whom there were no neurological deficits. In another patient requiring gastrostomy feeds long-term, significant brain injury and persistent hyperinsulinism were noted. A summary of feeding problems for all 25 patients is included in Table 1.

**Table 1:** Clinical characteristics of disease and feeding problems.

	**Mutation**	**Type**	**Surgery**	**PN**	**Vomiting**	**Neurodev problem?**	**Sucking problem?**	**Swallowing problem?**	**Feed reluctance?**	**Aversion?**	**IP feeding**	**D/c feeding**	**6 m feeding**	**12 m feeding**	**36 m feeding**
1	*K_*ATP*_*	Diffuse	None	Yes	Yes	Some	No	No	No	Yes	Oral	Oral	NGT	Gastrostomy	Gastrostomy
2	*K_*ATP*_*	Focal	Lesionectomy	No	Yes	None	No	No	No	Yes	Gastrostomy	Gastrostomy	Oral	Oral	Oral
3	*K_*ATP*_*	Diffuse	Pancreatectomy	Yes	Yes	None	Yes	No	No	No	Gastrostomy	Gastrostomy	Oral		
4	*GCK*	Diffuse	Pancreatectomy	Yes	Yes	Severe	No	Yes	No	No	Gastrostomy	Gastrostomy	Oral	Oral	Oral
5	*K_*ATP*_*	Focal	Lesionectomy	No	Yes	None	No	No	No	No	Oral	Oral	Oral	Oral	Oral
6	*K_*ATP*_*	Diffuse	Pancreatectomy	No	Yes	None	No	No	No	Yes	Gastrostomy	Gastrostomy	Oral	Oral	Oral
7	*K_*ATP*_*	Diffuse	Pancreatectomy	No	Yes	None	Yes	No	No	Yes	Gastrostomy	Gastrostomy	Oral	Oral	Oral
8	*K_*ATP*_*	Focal	Unsuccessful	Yes	Yes	Severe	No	No	No	Yes	Oral	Oral	Oral	Oral	Oral
9	None	Diffuse	None	Yes	Yes	None	No	Yes	No	No	NGT	Oral	Oral	Oral	Oral
10	None	Diffuse	None	No	Yes	None	No	No	No	Yes	NGT	NGT	NGT	Oral	Oral
11	None	Diffuse	None	Yes	Yes	None	Yes	No	No	No	NGT	Oral	Oral	Oral	Oral
12	None	Diffuse	None	No	No	None	No	No	Yes	Yes	Oral	Oral	Oral	Oral	Oral
13	*K_*ATP*_*	Focal	Lesionectomy	No	No	None	No	No	Yes	Yes	Oral	Oral	Oral	Oral	Oral
14	*K_*ATP*_*	Diffuse	Pancreatectomy	No	No	None	Yes	No	No	Yes	Gastrostomy	Gastrostomy	Oral		
15	*K_*ATP*_*	Focal	Unsuccessful	No	Yes	Some	No	No	Yes	Yes	Gastrostomy	Gastrostomy	Gastrostomy	Gastrostomy	Oral
16	None	Diffuse	None	Yes	Yes	Some	No	No	No	Yes	NGT	NGT	NGT	Oral	Oral
17	None	Diffuse	None	No	Yes	None	Yes	No	No	Yes	NGT	NGT	NGT	Oral	Oral
18	None	Diffuse	Pancreatectomy	No	No	None	No	No	Yes	No	Oral	Oral	Oral	Oral	Oral
19	*K_*ATP*_*	Diffuse	Pancreatectomy	Yes	Yes	Severe	Yes	No	No	Yes	Gastrostomy	Gastrostomy	Gastrostomy	Gastrostomy	Gastrostomy
20	None	Diffuse	None	No	Yes	Some	No	No	Yes	Yes	Oral	Oral	Oral	Oral	Oral
21	*K_*ATP*_*	Diffuse	Pancreatectomy	No	Yes	None	No	No	No	Yes	Gastrostomy	Gastrostomy	Gastrostomy	Gastrostomy	Gastrostomy
22	*K_*ATP*_*	Focal	Lesionectomy	No	Yes	None	No	No	No	Yes	Gastrostomy	Gastrostomy	Gastrostomy	Oral	Oral
23	None	Diffuse	None	No	No	Severe	No	No	Yes	Yes	Oral	Oral	Oral	Oral	Oral
24	None	Diffuse	None	No	Yes	None	No	No	Yes	No	NGT	NGT	Oral	Oral	Oral
25	None	Diffuse	None	No	Yes	None	No	No	Yes	No	Oral	Oral	Oral	Oral	Oral

*Important characteristics relating to CHI and feeding problems are listed for all patients individually. Unsuccessful, focal lesion identified on imaging but not identified operatively so no resection; NGT, nasogastric tube; IP, inpatient; D/c, at discharge; M, months; PN, parenteral nutrition; Neurodev, neurodevelopmental*.

### Predictive Factors for Resolution of Feeding Problems

We investigated predictive factors for time to resolution of feed aversion and time to resolution of all feeding problems as a composite outcome. To assess for confounding factors, we investigated the following variables for association with time to resolution of all feeding problems: successful lesionectomy or not, neurodevelopmental problems, gene mutation status, glucose infusion rate (as a marker of disease severity), use of glucagon, octreotide, PN and NGT/gastrostomy feeding. In Cox regression analysis (−2 Log Likelihood for the overall model = 27.925, *p* = 0.05) use of PN (*p* = 0.041), NGT/gastrostomy feeding (*p* = 0.011), successful lesionectomy (*p* = 0.015), and glucose infusion rate (*p* = 0.029) all showed a significant association with time to resolution of all feeding problems. All other factors (including adverse neurodevelopment) showed no significant association. We then assessed those significant factors in isolation to assess for a predictive relationship. Those showing no significant association in Cox regression were not analyzed further.

Mean (standard deviation, SD) days of recovery from feed aversion following discharge was more rapid in those who underwent lesionectomy compared with those who did not [82(38) vs. 777(744), *p* = 0.16]. In patients with diffuse CHI, the time (days) to resolution of feed aversion was similar between those who underwent subtotal pancreatectomy and those treated with conservative medical therapy [894 (725) vs. 791 (874), *p* = 0.87], strengthening the association of resolution of feed aversion with curative lesionectomy.

**Figure 3:**
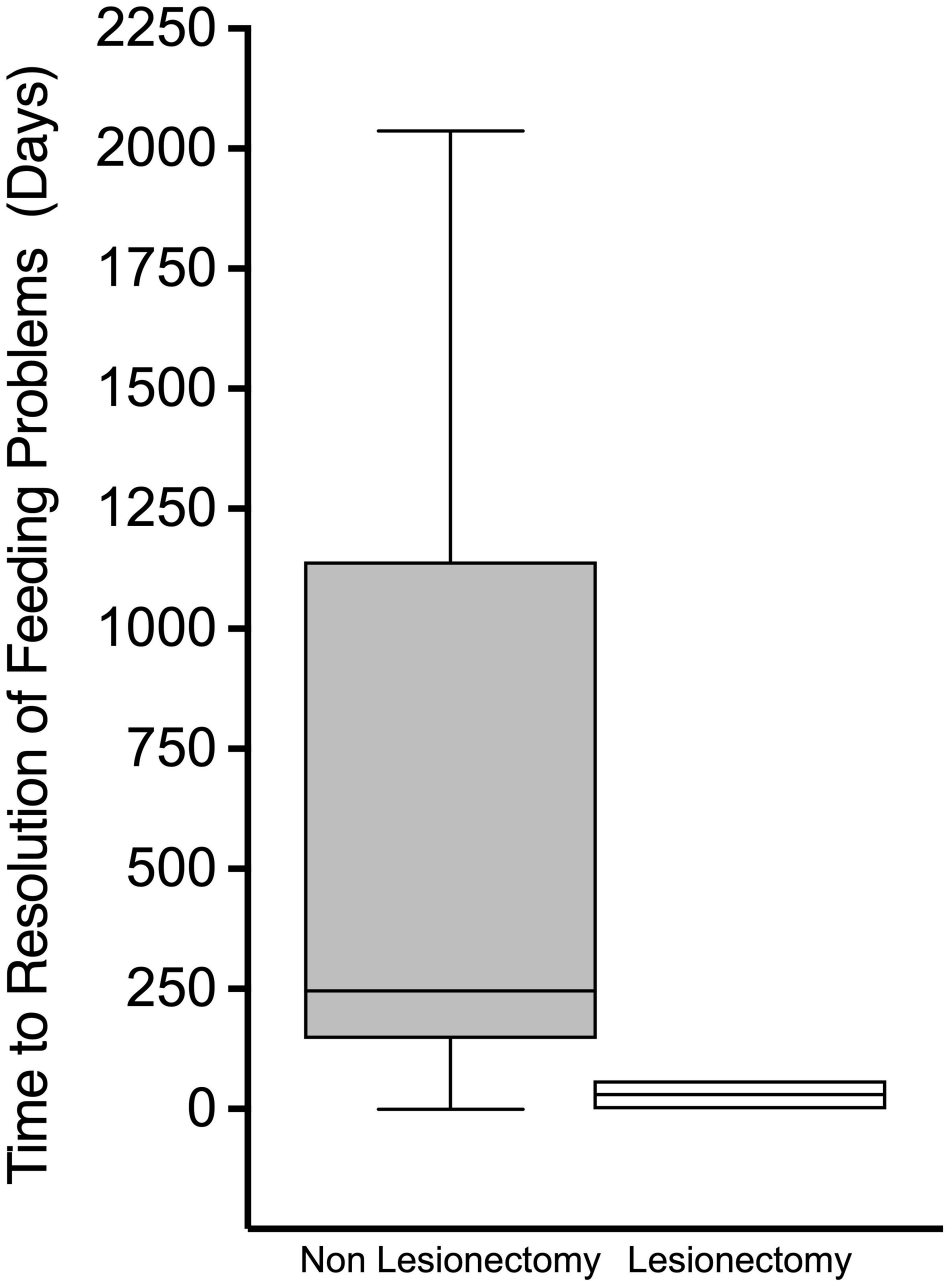
Box and whisker plot of time from discharge to resolution of feeding problems. Non-linear scale. Patients who had undergone lesionectomy demonstrated a much more rapid time from discharge to resolution of feeding problems.

When all feeding problems were considered as a composite outcome, time (days) from discharge to resolution was significantly lower in those who underwent lesionectomy than in those who did not [30(35) vs. 590(641), *p* = 0.009] (Figure 3), reinforcing the positive impact of cure of hyperinsulinism on feeding outcomes. In patients with diffuse CHI, time (days) to resolution of feeding problems was similar between those undergoing subtotal pancreatectomy and those treated by conservative medical therapy [767(692) vs. 454(666), *p* = 0.52], providing contrasting evidence of the impact of persistent hyperinsulinism on adverse feeding outcomes.

When each factor was assessed in isolation neither use of parenteral nutrition nor nasogastric tube feeding/gastrostomy feeding showed a significant difference in time to resolution of all feeding problems on Mann-Whitney *U* testing (*p* = 0.343 and *p* = 0.521, respectively).

Age at presentation had no correlation with time to resolution of feeding problems (Spearman Rank −0.138, *p* = 0.729). Maximum doses of Diazoxide (mg/kg/day) and Octreotide (mcg/kg/day) showed no correlation with time to resolution of feeding problems (Spearman Rank 0.148, *p* = 0.629 and Spearman Rank −0.081, *p* = 0.775, respectively). We examined hepatic ultrasound scans and liver function tests in all patients receiving octreotide but did not identify evidence for well-formed gall stones, cholecystitis, or persistent hepatic dysfunction. Despite significance in Cox regression analysis, glucose infusion rate (mg/kg/min) showed no correlation with time to resolution of feeding problems when assessed in isolation (Spearman Rank −0.20, *p* = 0.946).

In summary, the only factor that was significantly associated with time to resolution of all feeding problems in both Cox regression analysis and in isolation was whether or not the patient had undergone a lesionectomy.

## Discussion

We have provided the first description of feeding problems in a longitudinal cohort of children with CHI. Previous work undertaken by our group has described the risk factors for developing feeding problems in those with CHI (7) and while anecdotally feeding problems are common and problematic in many patients and their families, there are no studies describing the long term outcomes of patients with feeding problems in CHI. Our study has expanded on the longitudinal description of feeding problems, laying emphasis on the distressing subtype of feed aversion. While we find that feeding problems are frequent in CHI, resolution is possible, although delayed in many patients.

One strength of our study is that it combines objective diagnosis of feeding problems by specialists with parental reports, both at diagnosis and in follow-up. This combination is likely to reduce reporting bias and provide a true representation of the extent and duration of feeding problems in CHI. Our study is limited by the number of patients with feeding problems, particularly those undergoing successful lesionectomy and this is reflected in the associated non-significant statistical value for resolution of food aversion. However, for a specific outcome in a longitudinal cohort of a rare disease, the numbers of patients are large enough to derive reasonable inferences.

We have noted a relatively rapid reduction in some types of feeding problems, with amelioration of vomiting by two months after discharge from the initial admission. In contrast, feed aversion is more persistent and can continue for 2–3 years after initial recognition, in some cases requiring non-oral enteral feeding for the entire period. This difference in time to resolution is not surprising as vomiting was likely secondary to gastroesophageal reflux which frequently resolves spontaneously in infancy and is amenable to medical therapy. Conversely, feed aversion cannot be managed with medical therapies and would be unresponsive to pharmacologic interventions, instead requiring therapeutic behavioral interventions. We have not identified the precise factors prolonging feed aversion, but a complex interplay of multiple factors including disease severity, insulin lowering medication action and side effects, non-oral enteral feeding and interruption of normal feeding milestones are likely to be causative or associative (3).

The phenotype of rapid resolution of feeding problems following successful focal lesionectomy makes a compelling case for normalization of disease complexity and severity to improve feeding outcomes. However, complete resolution of disease also allows for cessation of medications and supraphysiological calorie intake both of which may have been contributing to feeding problems. Our study is unable to ascertain specific factors and pathways that enable swift resolution following cure from hyperinsulinism.

We have also described the initial rapid reduction in the number of patients requiring non-oral feeding after discharge followed by a plateau between 12 and 36 months. This pattern is not exclusive to CHI but common in feed aversion due to diverse causes.

Our findings are in keeping with a previous observation of feeding problem resolution in those undergoing successful pancreatic surgery (4) although it is not clear if study patients included both focal and diffuse CHI. Nonetheless, the strong correlation of amelioration of hyperinsulinism and hypoglycaemia with resolution argues for a role for hyperinsulinism and/or adjunctive and therapeutic factors in the pathogenesis of feeding problems in patients with CHI. The observation that endogenous insulin in hypothalamic feeding centers may play a role in rat models, together with patterns of persistence and resolution of feeding problems with hyperinsulinism (10, 11) suggests the possibility of a pancreatic/gut-hypothalamic axis that regulates feeding behavior; such models require further exploration in animal models and larger cohorts of patients with CHI.

## Conclusions

A significant proportion of patients with CHI have feeding problems identified at the time of initial hospital admission. Some forms of feeding problems such as feed aversion are slow to resolve and may persist for more than three years. In contrast, feeding problems resolve more rapidly in those with focal CHI undergoing curative lesionectomy suggesting the influence of persistent hyperinsulinism on adverse feeding outcomes.

## Data Availability Statement

The datasets generated for this study are available on request to the corresponding author.

## Ethics Statement

The studies involving human participants were reviewed and approved by Royal Manchester Children's Hospital REC/H1010/88. Written informed consent from the participants' legal guardian/next of kin was not required to participate in this study in accordance with the national legislation and the institutional requirements.

## Author Contributions

CW performed the retrospective data collection from patient notes, the research telephone clinics and all of the statistical analysis, wrote the manuscript and approved the final version. CH designed the data collection sheet for prospective data collection, assessed, and diagnosed patients with feeding problems, performed the prospective data collection, and reviewed and edited the manuscript and approved the final version. SW and NG assessed and diagnosed patients with feeding problems, performed the prospective data collection. EO performed the prospective data collection as well as retrospective data collection of drug dosages. IB and MS-E assessed and diagnosed patients with feeding problems as well as leading their care while an inpatient. MD contributed biochemical knowledge to the problem of feeding problems. NG, SW, EO, MS-E, MD, and IB reviewed and edited the manuscript and approved the final version.

### Conflict of Interest

The authors declare that the research was conducted in the absence of any commercial or financial relationships that could be construed as a potential conflict of interest.
